# Practical Considerations Relating to Routine Clinical Biomarker Testing for Non–small Cell Lung Cancer: Focus on Testing for *RET* Fusions

**DOI:** 10.3389/fmed.2020.562480

**Published:** 2021-01-21

**Authors:** Roy S. Herbst, Dara L. Aisner, Joshua R. Sonett, Andrew T. Turk, Joshua L. Weintraub, Neal I. Lindeman

**Affiliations:** ^1^Section of Medical Oncology, Department of Internal Medicine, Yale Cancer Center, Yale School of Medicine, New Haven, CT, United States; ^2^Department of Pathology, University of Colorado School of Medicine, Aurora, CO, United States; ^3^Division of Thoracic Surgery, Lung Transplant Program, Columbia University Medical Center, New York, NY, United States; ^4^Department of Pathology and Cell Biology, Columbia University, New York, NY, United States; ^5^Division of Interventional Radiology, Columbia University Irving Medical Center, New York, NY, United States; ^6^Department of Pathology, Brigham and Women's Hospital, Boston, MA, United States

**Keywords:** gene fusion, molecular testing, molecular pathology, NSCLC, RET

## Abstract

For patients with advanced non–small cell lung cancer, genomic profiling of tumors to identify potentially targetable alterations and thereby inform treatment selection is now part of standard care. While molecular analyses are primarily focused on actionable biomarkers associated with regulatory agency-approved therapies, there are a number of emerging biomarkers linked to investigational agents in advanced stages of clinical development will become approved agents. A particularly timely example is the reported data and US Food and Drug Administration approval of highly specific small molecule inhibitors of the proto-oncogene tyrosine-protein kinase receptor RET indicate that testing for tumor *RET* gene fusions in patients with NSCLC has become clinically important. As the number of biomarkers to be tested in NSCLC grows, it becomes increasingly important to optimize and prioritize the use of biopsy tissue, in order to both continue to allow accurate histopathological diagnosis and also to support concurrent genomic profiling to identify perhaps relatively uncommon genetic events. In order to provide practical expert consensus guidance to optimize processes facilitating genomic testing in NSCLC and to overcome barriers to access and implementation, a multidisciplinary advisory board was held in New York, on January 30, 2019. The panel comprised physicians involved in sample procurement (interventional radiologists and a thoracic surgeon), surgical pathologists specializing in the lung, molecular pathologists, and thoracic oncologists. Particular consideration was given to the key barriers faced by these experts in establishing institutional genomic screening programs for NSCLC. Potential solutions have been devised in the form of consensus opinions that might be used to help resolve such issues.

## Introduction

Most patients with non–small cell lung cancer (NSCLC) present with advanced stage unresectable disease requiring treatment with systemic therapies. Genomic profiling of tumors to identify potentially targetable alterations and/or eligibility for immune checkpoint inhibition has resulted in improved clinical outcomes in particular patient subgroups and is now part of standard care (1, 2). In NSCLC, molecular analyses focus on actionable biomarkers associated with regulatory agency-approved therapies, including activating mutations of *EGFR* and *BRAF* and chromosomal rearrangements resulting in *ALK, ROS1*, and *NTRK1–3* gene fusions. Recently the US Food and Drug Administration (FDA) has approved therapies targeting *RET* fusions, RETVMO (selpercatinib) and GAVRETO (pralsetinib) as well as *MET* exon 14-skipping variants, TABRECTA (capmatinib) in NSCLC. Additionally, emerging targets such as *HER2* insertions, and *KRAS* mutations add to the list of important biomarkers that may be included in testing protocols. Genomic alterations are almost exclusively present in non-squamous carcinomas, including in ~30% of adenocarcinomas overall when considering biomarkers with approved targeted therapies. Actionable activating alterations in proto-oncogenes commonly result in constitutive signaling through an intrinsic protein kinase, which drives oncogenesis. Specific inhibitors targeting the kinase products of such genes have now been synthesized and have either gained regulatory approval or are in clinical development.

**Figure 1:**
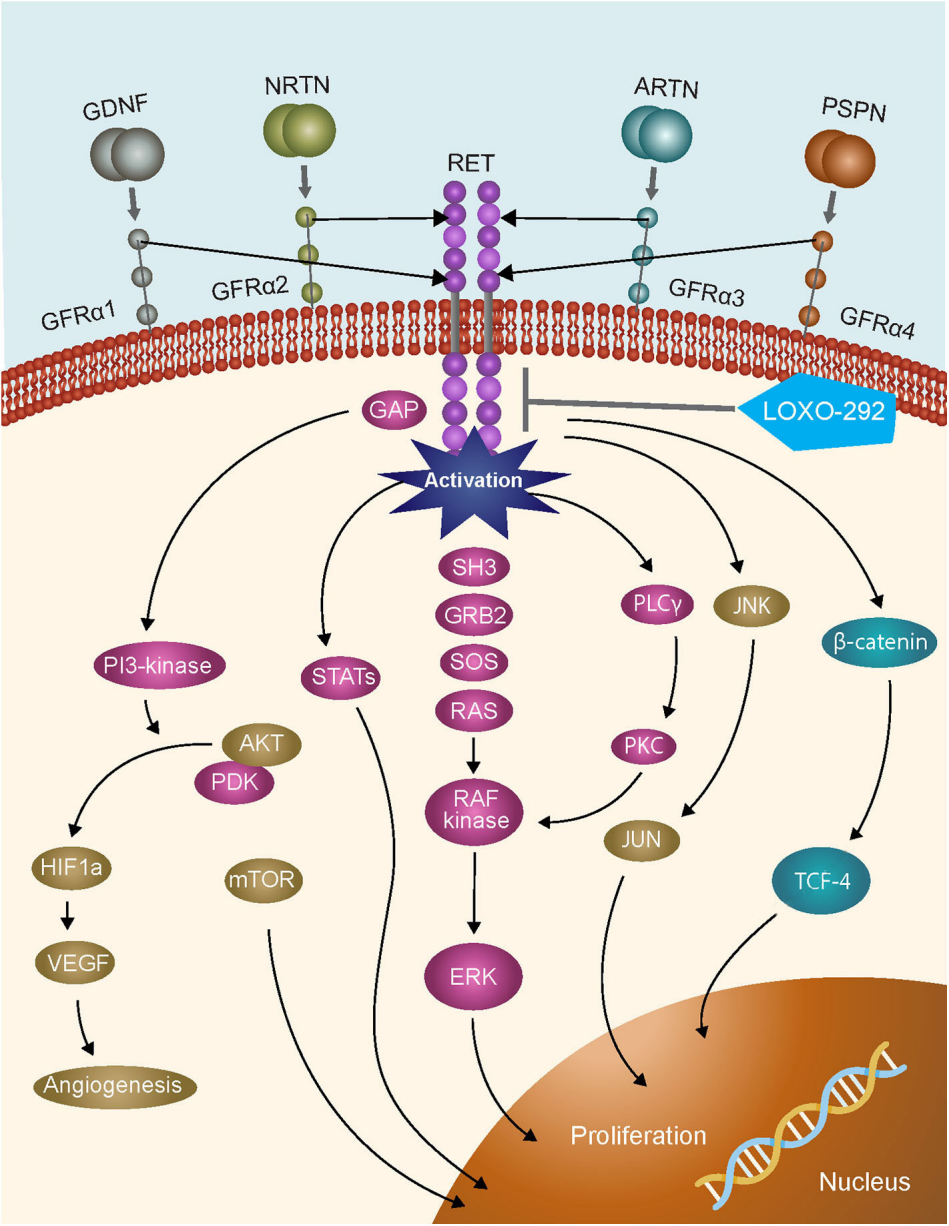
RET signaling pathways [adapted from (10)].

A timely example of the importance of expanded molecular testing in NSCLC are the recent FDA approvals of the specific RET inhibitors selpercatinib and pralsetinib which have resulted in a requirement to test for *RET* gene fusions in lung cancer, and activating point mutations in medullary thyroid cancer (3–7). The *RET* gene encodes the proto-oncogene tyrosine-protein kinase receptor RET, a transmembrane glycoprotein that plays a role in kidney and nervous system development, neuronal survival, and differentiation, and maintenance of spermatogonial stem cells (8, 9). RET activation results in the stimulation of multiple downstream signaling pathways, including RAS–MAPK and PI3K–AKT (Figure 1). Chromosomal rearrangements in tumors resulting in *RET* gene fusions are found in 1–2% of unselected patients with lung adenocarcinoma, and in 6–14% of adenocarcinomas wild-type for other known molecular drivers (11). Although predominantly found in adenocarcinomas, other NSCLC histological subtypes reportedly harboring *RET* gene fusions include large cell carcinoma (12), and adenosquamous carcinoma (13). A *RET* fusion has also been described in a squamous cell lung cancer, but the overall frequency in this histological subtype appears to be very low (14–16). Although outside of the scope of this review, *RET* fusions have also been identified in ~20% of papillary thyroid carcinomas (17, 18), and at a lower frequency (≤1%) in other tumor types including breast (19), colorectal (20), and pancreatic (21) cancers.

Clinical trials of multikinase inhibitors with activity against RET kinase have been shown to confer only modest benefits in patients with tumors harboring *RET* fusions, perhaps due to the toxicity associated with the concurrent inhibition of multiple kinase targets (8). Current guidelines consequently note that single gene testing for the presence of *RET* fusions is not recommended on a stand-alone basis in patients with advanced NSCLC, but suggest that *RET* analysis should form part of any large multigene panel test developed for patients with lung cancer (1). Based on emerging clinical data, several highly specific inhibitors of RET have recently been FDA approved. In particular, in a cohort of 105 patients with *RET* fusion-positive advanced NSCLC who had previously received platinum-based first-line chemotherapy, a response rate of 64% (95% CI: 54, 73%) was reported with the FDA approval of, selpercatinib, while a response rate of 85% (95% CI: 70%, 94%) was reported for treatment naive patients with *RET*-fusion positive advanced NSCLC treated with selpercatinib (6). A response rate of 57% (95% CI: 46, 68%) was reported for a second specific inhibitor, pralsetinib, in a smaller cohort of 87 patients with *RET* fusion-positive advanced NSCLC who had previously received platinum chemotherapy and 70% (CI 95%: 50, 86%) in treatment naive patients with *RET*-fusion positive advanced NSCLC (7). As we anticipate an increase in the importance of screening for *RET* fusion-positive non-squamous NSCLC with the regulatory approval of highly specific RET inhibitors, either using single gene or multigene tests, we have reviewed the practices that ensure optimal access to the entire spectrum of relevant biomarkers in NSCLC clinical samples.

Despite the clarity in guideline recommendations mandating certain biomarker testing in NSCLC (1, 2, 22, 23), and the definitive patient benefit associated with targeted agents as exemplified in the example of *RET* inhibitors, testing for these drivers may vary by geography and by institution. This can perhaps be most clearly seen in relation to *EGFR* testing (15, 24–26). A retrospective study of biomarker analyses across 15 US community oncology sites for patients with non-squamous NSCLC diagnosed between January 1, 2013 and December 31, 2015 revealed that only 59% of tumors had undergone genomic testing for *EGFR* and *ALK*, the two most well-established biomarkers, in accordance with the CAP/IASLC/AMP guidelines (24). This low rate of compliance might to some degree be explained by the timing of publication of the guidelines being during the study period in April 2013 (27), with endorsement by ASCO in October 2014 (28). The importance of identifying tumor drivers has been highlighted in a recent analysis of a longitudinal database of clinical data linked to genomic profiling results from routine care, which showed that for patients with driver mutations, overall survival was significantly longer for those who received targeted therapies compared with those who did not (18.6 vs. 11.4 months; *p* = 0.001) (29).

In order to provide practical expert consensus guidance to optimize genomic testing in NSCLC, in light of approved and developing clinically relevant biomarkers, and to overcome barriers to access and implementation, a multidisciplinary advisory board was held in New York, on January 30, 2019. The panel comprised physicians involved in sample procurement (interventional radiologists and a thoracic surgeon), surgical pathologists specializing in the lung, molecular pathologists and thoracic oncologists. Particular consideration was given to the key barriers faced by these experts in establishing institutional genomic screening programs for NSCLC. Potential solutions have been devised in the form of consensus opinions that might be used to help resolve such issues.

## Tissue Procurement Practices and Techniques

In order to maximize the level of biomarker testing, a shift in practice is required from a biopsy being viewed primarily as a tool for histological diagnosis to one where it is utilized as a route to complete pathologic staging, histological diagnosis, molecular profiling of the tumor, and ultimately a patient-specific treatment decision. This is of critical importance as unless molecular profiling is specified upfront and that message reaches the surgical pathologist and/or cytopathologist, then much or all of the available tissue may be used for diagnostic approaches, including immunohistochemistry. Therefore, close communication between the oncologist, surgeon, pulmonologist, interventional radiologist and pathologist is required during the entire specimen acquisition process.

Both fine needle aspirates (FNA) and percutaneous core biopsies can provide sufficient material for diagnosis and molecular profiling and both may be procured from the same procedure. Investigators have shown that these approaches have similar overall diagnostic value in relation to identifying lung malignancies in patients with lung lesions, with no significant difference in procedural complications (30). Likewise, it has been shown that these techniques are similarly effective in relation to providing adequate tissue for molecular evaluations (31). The maximum number of core biopsy samples obtained is limited by what can be safely done for the patient, with the understanding that being more aggressive at the first procedure has the very real potential to reduce the requirement for repeat procedures. In current practice, four to five 20 gauge or larger core biopsies are routinely obtained as an optimal number in institutions in which molecular testing is a clear expectation, as this allows for both diagnosis and molecular analysis. FNAs are commonly performed with a 20 gauge Chiba or Turner style needle. When possible, some investigators advise attempting both core biopsy and FNA sampling to maximize the possibility of obtaining a specimen that can provide sufficient material for molecular studies. The success of the cytology/pathology process is somewhat biased by local expertise as are the results of the published studies.

Consideration should also be given to which lesion to biopsy. In particular, those that can provide adequate tissue and best define the stage of the tumor should be chosen as the target lesions. An accessible metastatic site or suspicious mediastinal node would pathologically define the highest stage, while also generally providing sufficient tumor material for molecular profiling. The use of endobronchial ultrasound using FNA needles, if carefully performed, has been documented to provide adequate tissue for molecular and immunologic analyses (32, 33).

With regard to percutaneous biopsy, the size of the lesion is not the only consideration, but also the location in the lung. In patients with multiple bilateral lesions who have had prior thoracic surgery, the visceral and parietal pleura often scar together, reducing the chance of pneumothorax. Large, anteriorly located, peripheral pleural based, upper lobe lesions are perhaps the simplest to biopsy. The technique to avoid crossing multiple fissures which results in an increased rate of complications often requires placing patients in the prone position during the procedure. This is less well-tolerated compared with patients positioned supine. Peripheral lesions have been shown to decrease the incidence of crossing large central vessels that can result in intraparenchymal hemorrhage and hemoptysis. Anatomically, upper lobe lesions are preferred as the lung apices move less with respiration than lesions located near the diaphragm.

Lung biopsies have among the highest rate of complications of all radiologic procedures with the main associated adverse event being pneumothorax, reported in over 50% of patients in some series. However, the incidence of this potential complication can be considerably reduced through the use of a tract sealant system or blood patch (34–36). The vast majority of patients suffering from a pneumothorax can be treated with oxygen and observation. It is important to be aware that delayed pneumothorax can occur and post-procedure chest x-rays should be obtained after 2 h. Treatment of an uncomplicated pneumothorax may just require observation but may necessitate a chest tube/pigtail catheter if large or symptomatic. It should be kept in mind that the importance of an adequate biopsy far outweighs the risk of the procedure in patients without compromised performance status.

Other rarer complications arising from lung biopsies include hemoptysis, air embolization, tract seeding, and death. Tract seeding is exceedingly rare, with descriptions mostly limited to case reports; the reported frequency is 0.06% (37).

To quickly assess a core sample and make a determination not only as to whether diagnostic material is present but also to make a more extensive assessment of whether or not it is going to be adequate for molecular studies, rapid on-site evaluation (ROSE) is essential. In particular, ROSE can allow for the immediate designation of material for molecular analysis, minimizing the rate of tissue insufficiency and by extension, rebiospy (32). The methodology utilized for ROSE may also impact the suitability for testing, as in some cases, extensive touch-prep of a core biopsy may deplete the biopsy sample of tumor cells for subsequent testing. Thus, the approach for ROSE must be integrated with knowledge of the specimen types accepted by the laboratory. However, in community settings, ROSE may not be available due to lack of cytopathologist availability and the costs of providing such expertise.

In addition to cell block preparations from FNA procedures which are routinely utilized for molecular testing, many laboratories can also perform testing on cytological smears prepared and stained onsite which may improve chances for successful molecular diagnosis.

Although not informative in relation to anatomic pathology, for the purposes of molecular profiling, plasma-based cancer genotyping of circulating cell-free tumor (cf)DNA may provide an alternative to the analysis of NSCLC when, as indicated in current NCCN guidelines, a representative tumor tissue sample is not available for molecular studies (30, 38). However, despite high reported levels of specificity when compared with sequence analysis of tumor tissue, this approach still requires further development given the modest diagnostic sensitivity noted in some studies (39). Although identification of one of the aforementioned actionable biomarkers (i.e., *EGFR, BRAF, HER2, ALK, ROS1, RET, NTRK1–3, or MET*) or associated resistance mechanisms in cfDNA may be potentially informative, a negative plasma-based assay should be regarded as uninformative, with a chance of false negative results, and testing should be subsequently performed by a tissue-based analysis. The modest diagnostic sensitivity reported for cfDNA analyses is most likely a consequence of heterogeneous DNA shedding in patients with solid tumors, which may vary according to tumor stage and type. A more recent study has shown that the use of liquid biopsies may result in a greater number of biomarkers tested vs. current standard of care (sequential single biomarker tests) (38). However, specifically for fusion detection, further decreased sensitivity may be attributed also to the technical difficulty that may be associated with the DNA-based detection of such events and the incomplete probe coverage for many fusion events by commercially available cfDNA assays. This reduced sensitivity of fusion detection has been shown in several studies (40). On a practical level, bearing in mind the imperfect sensitivity of the approach and the potential need for a histologic diagnosis of NSCLC in some cases, if a cfDNA analysis is initiated, it may be advisable to schedule an interventional biopsy at the same time to avoid downstream delays in the event of uninformative cfDNA findings.

### Summary of Recommendations

- Both FNA and core needle biopsies may provide adequate tissue for histologic and molecular diagnosis, and both may be procured from the same procedure; 4–5 cores using a 20 gauge needle should be attempted to minimize the need for re-biopsy
- The most appropriate lesion for biopsy (whether in the lung or a metastatic lesion) should be chosen to allow for full staging and diagnosis while optimizing patient experience and minimizing patient adverse events
- While reportedly common and generally treatable, risk of pneumothorax can be significantly reduced by the use of tract sealants or a blood patch
- Onsite evaluation of the specimen by an experienced cytopathologist or equivalently trained professional can maximize tissue acquisition, improve diagnostic yield, aid in tissue triage for molecular studies and avoid re-biopsy
- cfDNA testing can be considered if tissue acquisition is infeasible but its limited sensitivity, particularly for fusions, must be considered

## Surgical Pathology/Cytology Workflow

**Figure 2:**
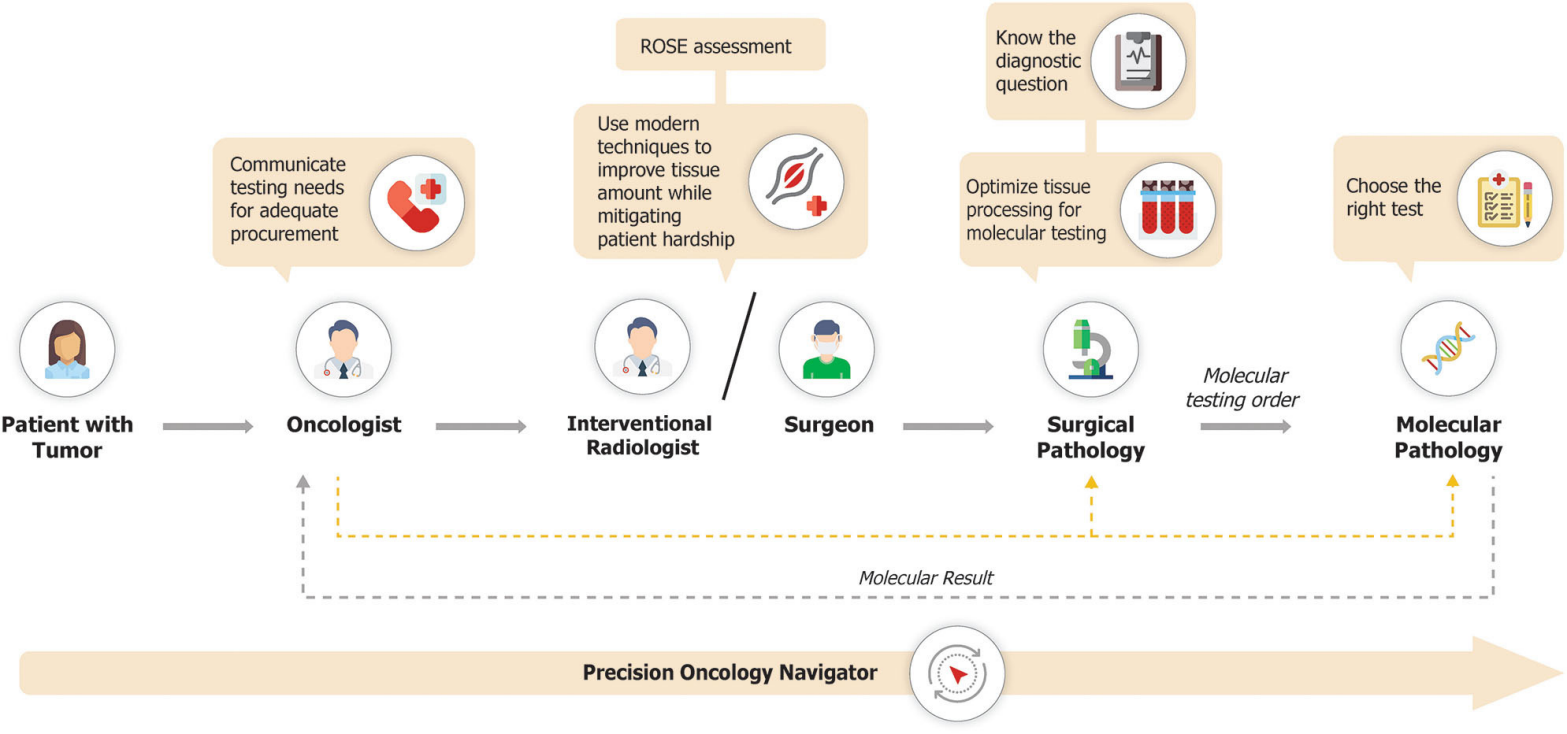
Precision oncology diagnostic workflow.

The precision oncology diagnostic workflow is summarized in Figure 2. Pathology laboratories typically receive large numbers of tissue samples each day. This creates the potential for those samples intended for molecular profiling to become incorporated into the mass of the daily histology workload, with the attendant risk that tissue is entirely used for histopathological diagnosis, leaving insufficient tissue for molecular analysis. If the tissue sample is for a new diagnosis, then histologic assessment is paramount. However, if it is from a patient with a prior histologic diagnosis and the main intention is the provision of a sample for molecular analysis, then minimal evaluation should be performed, to confirm that tumor is present and morphologically compatible with the known primary. To mitigate the risk of inadvertently exhausting the clinical sample, tissue for molecular investigations may need to be diverted into a system that facilitates molecular diagnosis. This can be achieved by establishing processes that allow for tissue samples to be clearly marked for molecular-prioritization workflows. Such samples can be routed to utilize minimal tissue to confirm the histologic diagnosis, with the bulk of the tissue then available for molecular analysis. In particular, consideration should be given to separately embedding each tissue biopsy from a patient into a single block rather than combining multiple cores into a single block. This allows for shallow facing of individual samples, which is generally sufficient to confirm the presence of tumor cells, while preserving the maximum amount of material for molecular analyses (41). Furthermore, a histology protocol which specifies that sections for molecular and immunohistochemical analyses are cut upfront with those for the morphological diagnosis may also contribute to tissue conservation, and is used by many laboratories. However, such procedures increase costs both in relation to the materials used such as cassettes, paraffin, and slides, and also in the requirement for more labor to cut the slides and process the blocks, and more space for the processors, with the alternative being that the turnaround time of the entire histology operation increases. The operational and cost impact of establishing specific workflows for tissue processing for molecular pathology will therefore vary depending on approach and will consequently need to be balanced with available resources.

### Summary of Recommendations

- Samples from patients with known and suspected NSCLC intended for molecular diagnosis should be prospectively identified within the histology workflow. This highlights the need for interdisciplinary communication at the time of sampling
- Once identified for molecular testing, specific processes to maximize tissue availability should be deployed, with the understanding that these may add cost and time for the providing laboratory and that constraints on the utilization of these processes may be reasonable

## Molecular Pathology Expertise and Testing Methodologies

Molecular testing in NSCLC can be carried out using single gene tests or can cover multiple genes by using targeted or broad-based assays, including next-generation sequencing (NGS) and 5′/3′ differential expression technology (Table 1). In addition, a sequential combination of both approaches can be used whereby key biomarkers are screened at the single gene level (e.g., *EGFR, ALK, ROS1*, and perhaps *KRAS* to exclude further testing and for clinical trial eligibility), and driver negative tumors are then subjected to broader NGS profiling. In general, single gene tests, such as polymerase chain reaction (PCR)-based assays for *EGFR* mutations (e.g., the Idylla platform by Biocartis), or fluorescence *in situ* hybridization (FISH) or immunohistochemistry for *ALK* fusions, can be carried out more quickly (i.e., 1–2 days for immunohistochemistry and 3–5 days for FISH), and are on an individual basis cheaper than NGS approaches. However, if multiple single marker assays are performed, the overall cost may exceed that of NGS analysis, which may consequently be more cost effective (42). In addition, carrying out multiple single gene assays may exhaust the available tissue before an actionable driver has been identified, especially as the number of relevant biomarkers for which to test continues to increase. Reported turnaround times for large, tissue-based gene panels generally range from 14 to 21 days. Some NGS-based assays can yield results in 7–14 days, although these are often highly targeted assays which may not detect the full spectrum of alterations seen in NSCLC. Although such analyses take a longer time than individual single gene tests, they can be used to simultaneously screen for large numbers of driver mutations with relevance across multiple indications more rapidly than sequentially performing multiple single gene tests. Moreover, for most laboratories, single gene tests are performed in batched analyses, often once per week, which introduces a “station-to-station” workflow when multiple different tests are needed for a given sample, even further extending the typical turnaround time in a sequential testing model. Factors affecting turnaround time require careful attention, given the necessity for rapid diagnosis, which is paramount in NSCLC therapeutic decision making. Pathologists at well-established laboratories are generally able to modulate logistical parameters in order to ensure efficient turnaround time. However, for patients treated in community oncology practices, where access to molecular testing capabilities may be limited, these factors may warrant consideration of send-out testing performed by regional or national reference laboratories.

**Table 1:** Molecular testing methods to detect *RET* and other gene fusions in NSCLC.

**Method**	**Pros**	**Cons**	**Recommendation**
IHC	Generally available Rapid turn-around-time Reimbursed	Significant false negatives and false positives	Not useful in detecting *RET* alterations due to low sensitivity and specificity
FISH	Generally available Rapid turn-around-time Reimbursed	High false positives and false negatives Requires significant validation efforts	Recommended if NGS or RT-PCR are not available
RT-PCR	Generally available Rapid turn-around-time Cheap	Limited to specified fusion partner detection Not commonly used in NSCLC workflow	Recommended, particularly if part of a multiplexed assay
5′/3′ differential expression	Multiplexable design Hybridization-based assay	Not commonly used in NSCLC workflow Requires significant validation efforts	Not recommended until more comparative data available
DNA-based NGS	Multiplexed and can detect SNVs as well as CNVs	Poor coverage of some intronic regions	Recommended, particularly as part of an RNA/DNA assay
RNA-based NGS	Unbiased fusion information without intron coverage issues	Highly dependent on RNA quality	Preferred method for fusion detection, including *RET*

*NSCLC, non–small cell lung cancer; IHC, immunohistochemistry; FISH, fluorescence in situ hybridization; RT-PCR reverse transcriptase polymerase chain reaction; NGS, next-generation sequencing; SNVs, single nucleotide variants; CNV, copy number variations*.

If samples have been processed appropriately, both DNA and RNA can be extracted from biopsies. This allows for DNA- and RNA-based NGS investigations, with the latter particularly important for the detection of certain targetable gene fusions, such as those involving *ROS1, NTRK2, NTRK3, NRG1*, and *RET* in particular. The recent tumor type agnostic approvals of larotrectinib and entrectinib for patients with tumors harboring *NTRK* gene fusions makes the detection of patients with TRK fusion NSCLC an emerging clinical priority (43, 44). RNA analysis is also highly effective when assessing splicing mutations, such as the *MET* exon 14 skipping mutations that may predict response to targeted inhibition of the MET kinase (45).

Access to biopsy tissue, even if sufficient for molecular testing, may be an additional barrier to overcome in some healthcare settings. As an example, a patient may have tissue procured at a local hospital and then be referred to an oncologist in another practice, who will order a set of molecular tests. The coordination between oncologist, the molecular laboratory and the tissue-holding hospital adds additional time, communication complexities and cost to the process. Some laboratories and healthcare systems have identified personnel resources to help smooth these workflow barriers.

### Summary of Recommendations

- While sequential single gene biomarker testing may be appropriate in certain clinical settings (e.g., urgent clinical decision making), the risk of tissue exhaustion and the overall cost effectiveness of NGS in NSCLC makes it the method of choice to screen tumors for actionable drivers
- Analysis of both DNA and RNA by NGS can improve sensitivity for fusions (e.g., *ROS1*) and splice alterations (e.g., exon 14 skipping mutations in *MET*)

## Analytical Considerations for *RET* Testing

The *RET* proto-oncogene is located at chromosome 10q11.21. The canonical transcript, as described in the UniProt Knowledgebase (46), is derived from 20 exons spanning a genomic region of ~53 kb, with the intrinsic tyrosine kinase encoded by exons 12–19. In gene fusions, the breakpoints in *RET* tend to be clustered in intron 11, which spans 1,847 base pairs, and is directly 5′ to the exons encoding the RET kinase function (47). In NSCLC, at least 15 different 5′ fusion partner genes have so far been identified, with this number likely to rise as tumors are increasingly profiled in research and routine healthcare settings using NGS. The most common *RET* 5′ fusion partners in NSCLC are *KIF5B* localized to 10p11.22 and *CCDC6* localized to 10q21.2 (48, 49). The chromosomal rearrangements creating *KIF5B-RET* and *CCDC6-RET* fusions are consequently pericentric and paracentric inversions of chromosome 10, respectively.

Techniques that have been used to detect or imply the presence of *RET* fusions in clinical samples include essentially single analyte methods such as FISH, immunohistochemistry, and conventional reverse transcriptase PCR (RT-PCR) analysis and those which can be used for highly multiplexed parallel assessments such as 5′/3′ differential expression assays and NGS.

Of the single analyte methods, immunohistochemistry (IHC) approaches reported to date appear to be of limited value in relation to the detection of *RET* fusions due to low sensitivity and highly variable specificity, when compared with molecular methods. Studies have shown that the sensitivity of *RET* IHC ranges between 55 and 65% with specificities of 40–85%, leading clinical guideline authors to recommend additional studies before consideration is given to the use of IHC for the identification of tumors harboring *RET* fusions (1, 11, 50). While conventional RT-PCR assays may give high sensitivity and specificity for specific gene fusion events when primers are cited in particular 5′ partner and *RET* exons located above and below expected breakpoints, such assays are limited to the detection of defined and characterized fusions, and will not detect rearrangements involving novel 5′ partners. In addition, RT-PCR analysis of NSCLC patient samples is not routinely integrated into pathology laboratory workflows, which may represent a barrier to implementation of an RT-PCR-based method in this clinical context. By contrast, break apart interphase FISH assays can detect *RET* fusions without knowledge of the partner gene, and FISH assays are standard diagnostic tools in pathology laboratories. However, their interpretation in the analysis of *RET* gene fusions can be complicated, and the lack of standardized criteria may lead to high false positive rates when compared with confirmatory NGS analyses. These discrepancies may be most commonly associated with the scoring of single 3′ signals in *RET* break apart assays as indicative of the presence of a fusion, when such signals perhaps instead reflect the fragility of the *RET* locus. In addition, because some *RET* chromosomal rearrangements are paracentric inversions, a higher probability of false-negative results is implicit, as such inversions, particularly when accompanied by interstitial deletions, are likely to result in lack of separation of FISH probes. Defining thresholds to avoid false negative calls for break apart FISH may be challenging, while optimizing specificity by decreasing false positives may be more straightforward. However, in the setting of a clinical need for rapid turnaround times for the diagnosis of a *RET* fusion, use of single biomarker assays such as FISH or RT-PCR may be required, if tissue allows.

A multiplex approach that may be used to identify *RET* and other fusions is to use a 5′/3′ differential expression assay (e.g., Nanostring technology) (51–55). As such assays use a hybridization-based design, enzymatic processes are not required, and consequently, they may be a more sensitive analytical tool than RT-PCR in clinical material yielding poor quality RNA, such as formalin-fixed paraffin-embedded samples (56). Assays can allow for a direct assessment of the presence of a particular fusion transcript if reporter and capture probes are positioned in exonic sequences of 5′ and 3′ partner genes either side of an expected breakpoint. Although readily multiplexable, this approach will only work for known and characterized fusions. Alternatively a measurement of the relative abundance of RNA sequence from 3′ and 5′ exons of a proto-oncogenic fusion partner such as *RET* can be used to imply the presence of a fusion without knowledge of the 5′ partner, followed by confirmation by an additional method (53, 54). However, until more comparative studies have been undertaken, the diagnostic potential for the routine clinical use of such approaches in the detection of *RET* fusions remains to be determined.

As increasing numbers of clinically relevant predictive biomarkers are characterized across multiple disease settings, it is likely that NGS will become the key diagnostic tool to inform treatment decisions in advanced cancers. NGS assays can be DNA- or RNA-based and can be targeted to a panel of genes relevant to treatment decisions, either through an amplicon or hybridization capture design. In relation to the detection of gene fusions, amplicon-based enrichment strategies require the 5′ partner gene and breakpoints to be known, whereas hybridization capture approaches allow for the detection of novel fusion partners. A more recent technology, anchored multiplexed PCR, combines these approaches, with a targeted capture at one end of the fusion RNA to provide specificity, and a second adapter ligated to the other end of randomly sheared nucleic acid strand, to enable the detection of all fusions, regardless of the specific partner.

Targeted designs maximize sequencing depth and sensitivity, and the information that can be extracted from limited tissue samples. If both DNA and RNA are extracted from a clinical sample, parallel NGS of both components allows for the detection of single nucleotide variants, small insertions or deletions, copy number variations, and chromosomal rearrangements. The analysis of RNA is particularly important for the detection of gene fusions that may have breakpoints within large repetitive element-rich introns, as is the case for *ROS1, NTRK2*, and *NTRK3* gene fusions, which are predictive of response to specific kinase inhibitors, including in NSCLC (57, 58). In the case of *RET* fusions in NSCLC, the genomic region in which breakpoints occur appears to be amenable to hybridization capture DNA-based NGS, given the sizes of the *RET* introns that tend to be involved (47). However, in a study by Benayed et al., a proportion of tumors deemed driver negative by DNA-based NGS were in fact positive for *RET* and other fusions when samples were subjected to RNA-based NGS (59). It is likely in the future therefore, that an optimal NSCLC diagnostic approach will involve the combined analysis of both DNA and RNA, so that all relevant predictive biomarkers can be assessed in one sample through DNA and RNA processes. Predictive NGS panels are already increasingly configured to allow for the comprehensive detection of biomarkers relevant to multiple separate indications. This will continue to facilitate rapid throughput of samples by avoiding indication specific batching of diagnostic NGS analyses.

### Summary of Recommendations

- Immunohistochemistry is of limited value currently in the detection of activating *RET* fusions
- FISH testing for *RET* fusions may be complicated by high false positive/false negative rates and should be used only after extensive validation and with recognition of appropriate circumstances in which to consider additional/orthogonal testing
- Hybridization capture DNA panels that are appropriately designed to detect *RET* fusions may be adequate for the detection of *RET* fusions
- Detection of fusions by RNA-based NGS, whether using an amplicon, hybridization capture or anchored multiplexed PCR (AMP) design are the recommended modalities for fusion detection in general, inclusive of *RET*, with the latter two being preferred

## Testing and Tissue Triage

It is likely that in a notable proportion of cases, the pathologist will conclude that the tissue available for analysis is insufficient to run all of the tests requested by the oncologist. In these situations, triage of biomarker testing may be required. With poorly differentiated lung tumors, often a substantial proportion of the available material is needed to investigate a large panel of potentially informative immunohistochemistry markers. In such situations it may be that procedures can be adjusted to balance diagnostic priorities with preservation of tissue for molecular analysis. For example, immunohistochemistry investigations may be ordered in sequential rounds and slides may be precut upfront. This may result in longer turnaround times in certain patients but may help preserve tissue for molecular analysis.

As a collaborative team, the pathologist, oncologist and/or the surgeon can assess the amount of tissue needed for each test and a decision can be made as to which tests should be done in the best interest of the patient, given their clinical history. In general, if a targetable driver mutation is identified, patients in the first instance are treated with an appropriate targeted agent, with immunotherapy reserved for second-line treatment. Available literature on the performance of immune checkpoint inhibitors as monotherapy in NSCLCs with known actionable driver mutations, including *RET* fusions, suggests a lack of efficacy of these agents in this setting (48, 60–62). The use of available biopsy material for biomarker testing should consequently reflect the available evidence and hierarchical treatment prioritization.

If there is insufficient tissue to complete the required number of molecular analyses, or in the event of disease progression, it may be necessary to re-biopsy the tumor, or supplement tissue-based investigations with plasma cfDNA analysis.

### Summary of Recommendations

- Close communication between pathologists and the treating oncologist is recommended to enable tissue triage for appropriate diagnostic testing in a tissue limited setting
- Individualized tissue triage should take into account the patient's clinical history and the most likely treatment regimen planned for that patient but genotyping of the tumor prior to initiation of treatment with an immune checkpoint inhibitor is recommended

## Communication and Coordination

There are many stakeholders in the tissue journey from procurement to treatment decision, and all need to remain involved in the process. The key therefore to establishing an effective biomarker testing program to facilitate treatment selection is to have good communication systems between the key functions. Potential barriers to effective communication include information technology infrastructure, geographical separation between functions such as procurement, pathology and oncology, the existence of cultural/departmental silos, and financial barriers to appropriate staffing.

As a first step to creating an effective process at an institutional level, there is a need to review the entire navigational path, from the patient's first visit through to molecular profiling and result reporting, to ensure that standardized mechanisms and communication points are established along this pathway. Dynamic maintenance of the established pathway will subsequently be required to overcome inevitable periodic breakdowns. Communication should therefore be thought of as an ongoing global management function, rather than as a series of interactions between individual experts. Drawing on the experience of clinical trial management, where study coordinators and navigators ensure proper specimen triage, we envisage a future state where such navigators are introduced into routine clinical practice. The appointed point person would have specific responsibility for interacting with relevant stakeholder departments and advocating adherence to a testing protocol that has been agreed upon by all clinical stakeholders. Importantly, we suggest that such navigators should not report to one department, but rather be an asset utilized across stakeholder departments and viewed as an advocate for the overall process. This could lead to improved adherence to clinical testing protocols in non-research settings. Funding of such an unorthodox position may be a barrier to implementation. More realistically, the identification of a physician advocate, perhaps within each relevant department, to motivate and coordinate testing would be an invaluable asset in the establishment of an effective evolving program. Finally, given the now rapid evolution and discovery of actionable driver mutations, each institution should have predefined time periods to reassess the measured targets and acquisition methods.

Tumor boards are used across tumor types, including in thoracic oncology to coordinate care between varying stakeholders involved in a cancer patient's care. The role of a molecular tumor board in a biomarker testing program has been described and may aid in improving coordination between stakeholders through careful selection of cases for testing (63).

### Summary of Recommendations

- A physician champion should be appointed to bridge technological, physical and departmental barriers and be an advocate for appropriate testing protocols
- Communication solutions between stakeholders should include a quality management system and be amenable to periodic adjustment
- Genomics navigators who can ensure a sample makes it from acquisition to report are recommended based on similar models used in clinical trials

## Issues Specific to the Community

Additional barriers to the establishment of an effective biomarker program may be present in community-based oncology practices and may be unique to this healthcare setting While limited data exist, such facilities may be restricted in the level of biomarker analyses that can be carried out onsite, and their access to expert molecular pathologists and interventional pulmonologists or interventional radiologists may be relatively limited. In the absence of established communication and coordination processes, as described herein, it is possible that sample collection may therefore be suboptimal and biopsy material may have to be sent out externally for biomarker analysis. If broad-based NGS analyses are requested in this situation using tissue or liquid samples, the complexity of reports may make them difficult to interpret by oncologists in such settings without local molecular pathology assistance. In addition, the increasing need for pathologists to manage/triage tissue for both diagnostic and molecular profiling can lead to increased costs and resource use, which may be difficult for smaller community-based laboratories to absorb. Such institutions may therefore benefit from educational opportunities providing specific technical guidance on how much tissue is required for NSCLC biomarker analysis, how to set up appropriately tailored systems, and how to build effective communication pathways for the interpretation of genomic data. A recent publication describing the establishment of a “virtual” molecular tumor board linking oncologists across the country to experts in molecular oncology may also highlight an opportunity for community practices without this specific expertise (64).

## Conclusions

It has now been widely established that for patients with NSCLCs harboring specific genetic lesions, appropriately targeted therapy improves treatment outcomes compared with standard chemotherapy. The number of actionable biomarkers is increasing, highlighted by recent FDA approval of two RET-fusion targeting agents selpercatinib and pralsetinib in RET-fusino positive advanced NSCLC patients. The list of biomarkers to test in advanced NSCLC populations includes not only those associated with approved agents, but also those associated with investigational agents. The amount of tissue procured, the processing of that tissue in the pathology laboratory and the methods employed for genotyping, as well as the triage of tissue to optimize diagnostic yield requires communication systems and coordination across stakeholders to define who gets tested and how. The recommendations that we have produced for NSCLC, including for *RET* fusion detection, may be extrapolated to other tumor type or tumor agnostic settings as the field of precision oncology continues to grow.

## Author's Note

The consensus recommendations described in this report are the outcome of the deliberations of the members of an expert panel convened at a meeting organized by Loxo Oncology, Inc., a wholly owned subsidiary of Eli Lilly and Company. The panel comprised RH, DA, JS, AT, JW, and NL, in addition to Harvey Pass MD and David Carbone MD Ph.D.

## Author Contributions

RH, DA, JS, AT, JW, and NL contributed to the writing of this manuscript. All authors contributed to the article and approved the submitted version.

## Conflict of Interest

RH reports grants and personal fees from AstraZeneca, Eli Lilly and Company, and Merck and Company; personal fees and other support in relation to scientific advisory boards from Infinity Pharmaceuticals, Neon Therapeutics, and NextCure; personal fees and other support in relation board membership (non-executive/independent) from Junshi Pharmaceuticals; and personal fees from Abbvie Pharmaceuticals, ARMO Biosciences, Biodesix, Bolt Biotherapeutics, Bristol-Myers Squibb, EMD Serrano, Genentech/Roche, Genmab, Heat Biologics, Halozyme, IMAB Biopharma, Immunocore, Loxo Oncology, Midas Health Analytics, Mirati Therapeutics, Nektar, Novartis, Pfizer, Sanofi, Seattle Genetics, Shire PLC, Spectrum Pharmaceuticals, Symphogen, Takeda, Tesaro, Tocagen, outside the submitted work. DA reports personal fees in relation to consultancies from Bayer Oncology, Genentech, AbbVie, and Bristol Myers Squibb, outside the submitted work. JS reports personal fees from Loxo Oncology in relation to advisory board attendance, during the conduct of the study. AT reports personal fees from Loxo Oncology in relation to advisory board attendance, during the conduct of the study. The remaining authors declare that the research was conducted in the absence of any commercial or financial relationships that could be construed as a potential conflict of interest.
